# Mortality and pathology in birds due to *Plasmodium (Giovannolaia) homocircumflexum* infection, with emphasis on the exoerythrocytic development of avian malaria parasites

**DOI:** 10.1186/s12936-016-1310-x

**Published:** 2016-05-04

**Authors:** Mikas Ilgūnas, Dovilė Bukauskaitė, Vaidas Palinauskas, Tatjana A. Iezhova, Nora Dinhopl, Nora Nedorost, Christiane Weissenbacher-Lang, Herbert Weissenböck, Gediminas Valkiūnas

**Affiliations:** Nature Research Centre, Akademijos 2, LT-08412 Vilnius, Lithuania; Institute of Pathology and Forensic Veterinary Medicine, University of Veterinary Medicine Vienna, 1210 Vienna, Austria

**Keywords:** Avian malaria, *Plasmodium homocircumflexum*, Virulence, Exoerythrocytic development, Phanerozoites

## Abstract

**Background:**

Species of avian malaria parasites (*Plasmodium*) are widespread, but their virulence has been insufficiently investigated, particularly in wild birds. During avian malaria, several cycles of tissue merogony occur, and many *Plasmodium* spp. produce secondary exoerythrocytic meronts (phanerozoites), which are induced by merozoites developing in erythrocytic meronts. Phanerozoites markedly damage organs, but remain insufficiently investigated in the majority of described *Plasmodium* spp. Avian malaria parasite *Plasmodium* (*Giovannolaia*) *homocircumflexum* (lineage pCOLL4) is virulent and produces phanerozoites in domestic canaries *Serinus canaria,* but its pathogenicity in wild birds remains unknown. The aim of this study was to investigate the pathology caused by this infection in species of common European birds.

**Methods:**

One individual of Eurasian siskin *Carduelis spinus*, common crossbill *Loxia curvirostra* and common starling *Sturnus vulgaris* were exposed to *P. homocircumflexum* infection by intramuscular sub-inoculation of infected blood. The birds were maintained in captivity and parasitaemia was monitored until their death due to malaria. Brain, heart, lungs, liver, spleen, kidney, and a piece of breast muscle were examined using histology and chromogenic in situ hybridization (ISH) methods.

**Results:**

All exposed birds developed malaria infection, survived the peak of parasitaemia, but suddenly died between 30 and 38 days post exposure when parasitaemia markedly decreased. Numerous phanerozoites were visible in histological sections of all organs and were particularly easily visualized after ISH processing. Blockage of brain capillaries with phanerozoites may have led to cerebral ischaemia, causing cerebral paralysis and is most likely the main reason of sudden death of all infected individuals. Inflammatory response was not visible around the brain, heart and muscle phanerozoites, and it was mild in parenchymal organs. The endothelial damage likely causes dysfunction and failure of parenchymal organs.

**Conclusion:**

*Plasmodium homocircumflexum* caused death of experimental passerine birds due to marked damage of organs by phanerozoites. Patterns of phanerozoites development and pathology were similar in all exposed birds. Mortality was reported when parasitaemia decreased or even turned into chronic stage, indicating that the light parasitaemia is not always indication of improved health during avian malaria. Application of traditional histological and ISH methods in parallel simplifies investigation of exoerythrocytic development and is recommended in avian malaria research.

## Background

Avian malaria parasites of the genus *Plasmodium* (Haemosporida, Plasmodiidae) are widespread on all continents, except Antarctica [1, 2]. These parasites have complex life cycles [3, 4]. Sporogony occurs in numerous species of blood-sucking mosquitoes belonging to different genera of the Culicidae, which transmit avian malaria [1, 4–6]. The following development occurs in vertebrate hosts. Susceptible birds get infected when mosquitoes inject sporozoites during their blood meal. The sporozoites develop into the first generation of primary exoerythrocytic meronts (cryptozoites), which are found in the reticular cells of the skin and some other organs. Merozoites developing in cryptozoites cannot infect red blood cells, but induce the second generation of primary exoerythrocytic meronts (metacryptozoites), which develop in macrophages and other reticular cells in many internal organs and tissues. Merozoites developing in metacryptozoites are able to infect red blood cells. Part of merozoites from metacryptozoites invade erythrocytes and develop into erythrocytic meronts and gametocytes, while another part induces the next generations of metacryptozoites. Part of merozoites from the erythrocytic meronts along with part of merozoites developed in the metacryptozoites penetrate the endothelial cells of the capillaries and other reticular cells in many organs, initiating formation of secondary exoerythrocytic meronts (phanerozoites) [4, 7].

Detection of exoerythrocytic meronts using traditional histology methods is often difficult in tissue samples of naturally infected birds due to light infection of organs and difficulties to sample in wildlife sick individuals. Moreover, fragmented nuclei within necrotic tissues of sampled dead birds might be erroneously considered as exoerythrocytic meronts [8]. To overcome this diagnostic difficulty, chromogenic in situ hybridization (ISH) protocol was developed for detection of avian *Plasmodium* spp. [8]. Application of this method simplifies research on pathology caused by *Plasmodium* spp. and provides new data for better understanding of pathogenicity of avian malaria in wild birds [9].

More than 50 species of avian malaria parasites have been described, and their number is increasing [10–14]. However, the majority of recent studies deal mainly with the morphology of blood stages, molecular characterization, evolutionary biology, and distribution of these organisms. A few recent studies address exoerythrocytic development of avian *Plasmodium* spp. [9, 15, 16], but this information is crucial for better understanding pathological events during malaria infections.

Recently, a new *Plasmodium* species*, Plasmodium* (*Giovannolaia*) *homocircumflexum* (cytochrome *b* gene lineage pCOLL4) was described [14]. This parasite was isolated from a wild-caught red-backed shrike *Lanius collurio*, and experimental studies showed that this infection is often lethal in domestic canaries *Serinus canaria* due to marked pathology caused by phanerozoites. This parasite isolate was cryopreserved and is available for experimental research at the Nature Research Centre, Vilnius, Lithuania. However, there is no information about its virulence in wild birds, the aims of this study were: (1) to investigate effects of *P. homocircumflexum* (pCOLL4) on three species of common European birds (Eurasian siskin *Carduelis spinus*, common crossbill *Loxia curvirostra* and common starling *Sturnus vulgaris*); (2) to investigate dynamics of parasitaemia and development of phanerozoites in exposed birds; and, (3) to compare sensitivity of traditional histological and ISH methods in detection of tissue stages of avian malaria parasites.

## Methods

### Study site and experimental design

Birds were caught using mist nets and big funnel traps [17], and experiments were carried out at the Biological Station of the Zoological Institute of the Russian Academy of Sciences on the Curonian Spit in the Baltic Sea (55°05′ N, 20°44′ E) between 23 May and 16 July, 2014. The birds were exposed to experimental *P. homocircumflexum* (pCOLL4) infection and kept until they died. Blood samples and organs were collected and examined for blood stages and phanerozoites.

Juvenile Eurasian siskin, common crossbill and common starling were chosen for this research because they are abundant in Europe and are easy to maintain in captivity. *Plasmodium homocircumflexum* has not been reported in any of the three species of birds used in this study, so these birds can be considered as abnormal (non-adapted) hosts for this infection. It is worth noting that the range of the red-backed shrike and the experimental birds overlap in nature and transmission of malaria among them is theoretically possible.

The Eurasian siskin and the common crossbill were kept indoors in a vector-free room. The common starling was kept outside in a cage covered with a cover made of fine-mesh bolting silk, which prevented penetration of blood-sucking insects in the cage. All birds were kept at a natural light–dark photoperiod. Before experiments, they were examined for possible presence of natural infections by microscopic examination of blood films and later by polymerase chain reaction (PCR)-based methods (see description below). All birds were non-infected with haemosporidian parasites before the experiment.

The isolate of *P. homocircumflexum* (lineage pCOLL4, GenBank accession no. KC884250), which was originally obtained from a naturally infected red-backed shrike was used. This isolate was cryopreserved and is available at the P. B. Šivickis Laboratory of Parasitology, Nature Research Centre, Vilnius, Lithuania [14]. One sample of this isolate (parasitaemia intensity of 4 %) was thawed and used to infect one Eurasian siskin, as described by Palinauskas et al. [18] with slight modifications. Briefly, the frozen tube containing infected blood was thawed and mixed with 12 % NaCl (one-third of the thawed sample amount). After equilibration for 5 min at room temperature, one volume of 1.6 % NaCl was added, followed by centrifugation at 200 g for 5 min. After centrifugation, the supernatant was removed and 1.6 % NaCl (one-third of the original sample) was added and centrifuged again. After removing the supernatant, the same procedure was repeated three times with 0.9 % NaCl solution. The final mixture was diluted with 0.9 % NaCl and sub-inoculated into one Eurasian siskin, as described by Palinauskas et al. [18].

One common crossbill and one common starling were infected using blood (parasitaemia intensity of 50 %) collected from the exposed Eurasian siskin. Briefly, the brachial vein of the donor Eurasian siskin was punctured using a needle. About 100 µl of blood was mixed with 25 µl sodium citrate and 125 µl 0.9 % saline solution. The mixture was sub-inoculated into the pectoral muscle of the experimental birds, as described by Palinauskas et al. [18]. Six wild caught non-infected Eurasian siskins were used as controls. The birds were maintained in the same room as the experimental birds.

**Fig. 1:**
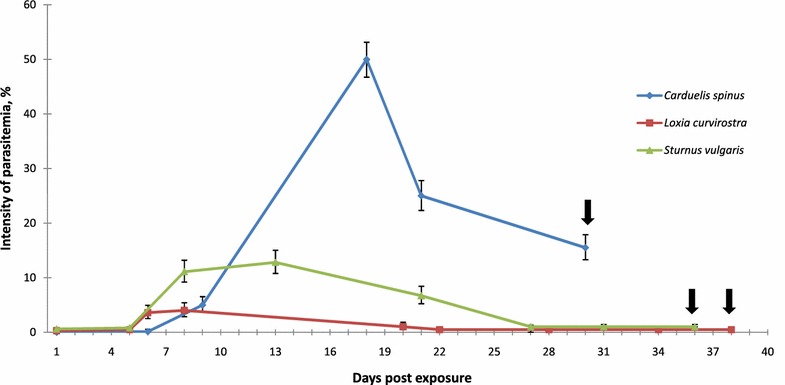
Dynamics of parasitaemia of *Plasmodium (Giovannolaia) homocircumflexum* (cytochrome *b* lineage pCOLL4) in the experimentally infected Eurasian siskin *Carduelis spinus*, common crossbill *Loxia curvirostra* and common starling *Sturnus vulgaris.* One individual bird of each species was exposed. *Black arrows* indicate days of bird deaths. *Vertical lines*: 95 % confidence limits of parasitaemia intensity

All birds were observed until the experimental birds died [30–38 days post exposure (dpe)]. Blood was taken for microscopic examination and PCR-based testing during the course of the experiment (see Fig. 1). Approximately 50 µl of blood was collected in heparinized microcapillaries by puncturing the brachial vein. A drop of blood was used to make three blood films, which were air-dried, fixed with absolute methanol, stained with Giemsa and examined microscopically as described by Valkiūnas et al. [19]. Approximately 35 µl of the blood left in the capillary was fixed in non-lysis SET buffer (0.05 M Tris, 0.15 M NaCl, 0.5 M EDTA, pH 8.0) for molecular analysis; these samples were kept at room temperature in the field and at −20 °C in the laboratory.

Brain, heart, kidney, liver, lungs, spleen, and a piece of the pectoral muscle of the experimental birds were dissected after the birds’ death. The samples were fixed in 10 % neutral formalin and embedded in paraffin blocks. Histological sections of 4 µm were prepared, stained with haematoxylin-eosin (H&E) [4] and examined microscopically.

### Morphological analysis

An Olympus BX51 light microscope equipped with Olympus DP12 digital camera and imaging software Olympus DP-SOFT was used to examine slides and prepare illustrations. To trace the parasitaemia, each blood slide was examined for 15–20 min at medium magnification (×400), and then at least 100 fields were studied at high magnification (×1000). Intensity of parasitaemia was estimated as a percentage by actual counting of the number of parasites per 1000 erythrocytes or per 10,000 erythrocytes if infections were light [20]. Microscopic examination was also used to determine possible presence of co-infections with other haemosporidian parasites. Histological preparations were examined using similar protocol, but they were also examined at low magnification (×200) for 10–15 min, followed by examination of the preparations for 10–15 min at medium magnification (×400) and then for another 20–30 min at high magnification (×1000). Statistical analyses were carried out using the ‘Statistica 7’ package.

### Molecular analysis

Total DNA was extracted from blood samples using the standard ammonium-acetate protocol [21] with a minor modification: instead of 250 µl of fixed blood, 125 µl was used. A nested-PCR protocol [22] was applied for the molecular analysis. For the first PCR, the primer pair HaemFNI [5′-CATATATTAAGAGAAITATGGAG-3′] and HaemNR3 [5′-ATAGAAAGATAAGAAATACCATTC-3′] was used. This is a general primer pair that amplifies the mitochondrial cytochrome *b* (cyt *b*) gene of *Plasmodium*, *Haemoproteus* and *Leucocytozoon* species. The reaction mix for the first PCR consisted of 12.5 µl of Dreamtaq Master Mix (Fermentas, Lithuania), 8.5 µl of nuclease-free water, 1 µl of each primer and 2 µl of template DNA. The thermal conditions for the first PCR were according to Helgren et al. [22]. For the second PCR, the primer pair HAEMF [5′-ATGGTGCTTTCGATATATGCATG-3′] and HAEMR2 [5′-GCATTATCTGGATGTGATAATGGT-3′] was used. This primer pair amplifies a 479 bp fragment of cyt *b* gene [23]. The reaction mix for the second PCR was identical to the mix for the first PCR, only instead of extracted DNA, 2 µl of the first PCR products were used as templates. The conditions for the second PCR were the same as in the first PCR, but 35 cycles instead of 20 were run. Success of the amplification was evaluated by running 2 µl of the second PCR product on 2 % agarose gel. One negative control (nuclease-free water) and one positive control (a *Plasmodium* sample, which was positive by microscopic examination of blood films) were used to determine possible false amplifications. No case of false amplification was found.

The cyt *b* gene fragments were sequenced from the 5′ end using the HAEMF primer [23]. Dye terminator cycle sequencing (Big Dye) was used. Samples were loaded onto an ABI PRISM TM 3100 capillary sequencing robot (Applied Biosystems, USA). Sequences of parasites were edited and examined using the BioEdit program [24]. The ‘Basic Local Alignment Search Tool’ and the megablast algorithm were used to identify the cyt *b* lineages of detected DNA sequences.

### In situ hybridization

Chromogenic in situ hybridization ISH was carried out according to Dinhopl et al. [8]. In brief, 3 μm paraffin wax-embedded tissue sections were subjected to proteolytic treatment with proteinase K (Roche, Basel, Switzerland) 6 μg/ml in Tris-buffered saline at 37 °C for 50 min. For hybridization, the slides were incubated overnight at 40 °C with hybridization mixture and a final probe concentration of 100 ng/ml. The used oligonucleotide probe (sequence: 5′-TTTAATAACTCGTTATATATATCAGTGTAGCAC-3′) was labelled with digoxigenin at the 3′ end (Eurofins MWG Operon, Ebersberg, Germany). The probe is aimed at 18S rRNA strand and is specific to detect avian *Plasmodium* spp. [9]. The digoxigenin-labelled hybrids were detected by incubating the slides with antidigoxigenin-AP Fab fragments (Roche) (1:200) for 1 h at RT. Visualization of the reaction was carried out using the colour substrates 5-bromo-4-chloro-3-indolyl phosphate (BCIP) and 4-nitro blue tetrazolium chloride (NBT) (Roche). Probe specificity has been extensively tested previously [8]. Tissues from a deceased wild Blackbird *Turdus merula* free of avian malaria parasites as well as application of an irrelevant oligonucleotide probe (designed for *Leishmania* spp.) on the experimental samples [see 8] were used as controls in this study.

### Ethical statement

Experimental procedures of this study were approved by the International Research Co-operation Agreement between the Biological Station Rybachy of the Zoological Institute of the Russian Academy of Sciences and Institute of Ecology of Nature Research Centre (25-05-2010). All efforts were made to minimize handling time and potential suffering of animals. None of the experimental birds suffered apparent injury during experiments.

## Results

All control birds survived and remained non-infected during this study. The exposed Eurasian siskin, common crossbill and common starling got infected, and PCR-based testing confirmed presence of the parasite lineage pCOLL4 in these birds.

According to microscopic examination of blood films, the prepatent period was 6, 6 and 8 dpe in Eurasian siskin, common crossbill and common starling, respectively. Parasitaemia remained during entire observation time (Fig. 1). The highest parasitaemia developed in the Eurasian siskin, and it reached 50 % on 18 dpe. Maximum parasitaemia was 4 % (8 dpe) and 12.8 % (13 dpe) in the common crossbill and common starling, respectively. The intensity of parasitaemia markedly decreased in all exposed birds between 20 and 27 dpe.

All exposed birds survived the acute parasitaemia stage, but suddenly died when parasitaemia decreased and was 15.5, 0.2, 0.1 % in the Eurasian siskin, common crossbill and common starling, respectively; these birds died 30, 38 and 36 dpe, respectively (Fig. 1). The mortality was sudden: all birds looked healthy in the evening, but were found dead the next morning. A marked enlargement of the spleen and liver, as well as cardiomegaly with dark pericardial effusion was recorded at post-mortem examination.

**Fig. 2:**
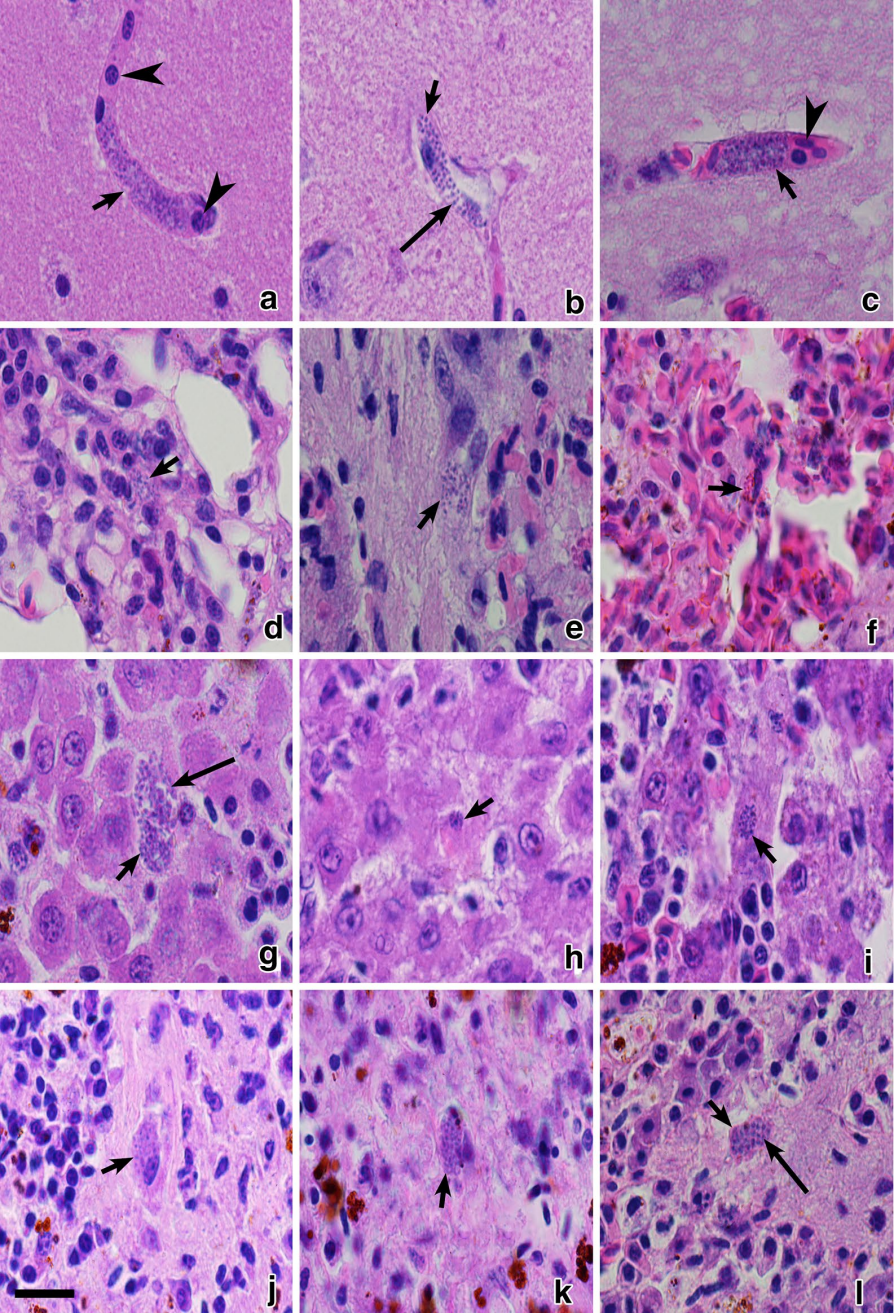
Phanerozoites of *Plasmodium (Giovannolaia) homocircumflexum* (cytochrome *b* lineage pCOLL4) in histological sections of brain (**a**–**c**), lungs (**d**–**f**), liver (**g**–**i**), spleen (**j**–**l**) of experimentally infected Eurasian siskin *Carduelis spinus* (**a**, **d**, **g**, **j**), common crossbill *Loxia curvirostra* (**b**, **e**, **h**, **k**) and common starling *Sturnus vulgaris* (**c**, **f**, **i**, **l**). *Short arrows*: phanerozoites, *long arrows*: merozoites, simple *arrowheads*: red blood cells in brain capillaries. Haematoxylin-eosin stained preparations. *Scale bar* 20 μm

Phanerozoites were observed in all dead birds. Numerous phanerozoites were detected in the brain capillaries (Fig. 2a–c); the parasites were large elongate bodies (maximum length is 34.6 µm), which followed shape of the brain capillaries. The phanerozoites blocked the capillaries resulting in the interruption of local circulation (Fig. 2a, c), which likely was the main reason of mortality. Numerous developing merozoites of roundish shape were readily visible inside phanerozoites (Fig. 2a–c). Inflammatory response was not visible around the brain phanerozoites.

**Fig. 3:**
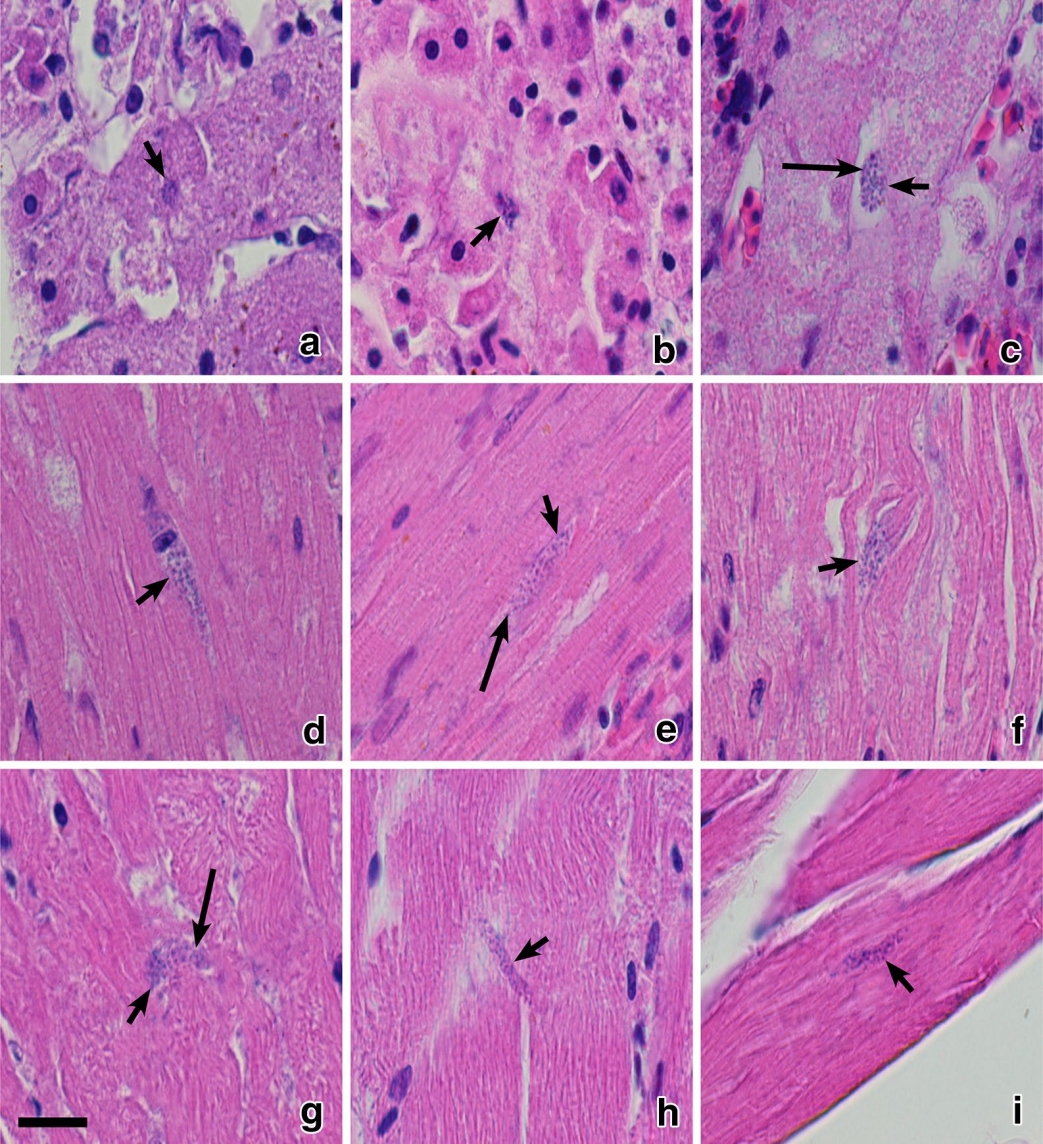
Phanerozoites of *Plasmodium (Giovannolaia) homocircumflexum* (cytochrome *b* lineage pCOLL4) in histological sections of kidney (**a**–**c**), heart (**d**–**f**) and pectoral muscle (**g**–**i**) of experimentally infected Eurasian siskin *Carduleis spinus* (**a**, **d**, **g**), common crossbill *Loxia curvirostra* (**b**, **e**, **h**) and common starling *Sturnus vulgaris* (**c**, **f**, **i**). *Short arrows*: phanerozoites, *long arrows*: merozoites. Haematoxylin-eosin stained preparations. *Scale bar* 20 μm

Phanerozoites were also numerous in lungs (Fig. 2d–f), liver (Fig. 2g–i), spleen (Fig. 2j–l), kidney (Fig. 3a–c), heart (Fig. 3d–f), and pectoral muscle (Fig. 3g–i) of all dead birds. In these organs, the parasites appeared as roundish or oval bodies developing in endothelial cells of capillaries and in macrophages. The inflammatory response was mild in parenchymal organs; it included lymphocytes, plasma cells, heterophils, and macrophages. The endothelial damage likely causes dysfunction and failure of parasitized organs.

In the heart (Fig. 3d–f) and breast muscles (Fig. 3g–i), phanerozoites were seen in endothelial cells of capillaries. The biggest parasites reached 28.6 µm in their large diameter; they were elongated, being similar in shape to the parasites observed in the brain. Inflammatory response was not visible in the heart or the breast muscles.

**Fig. 4:**
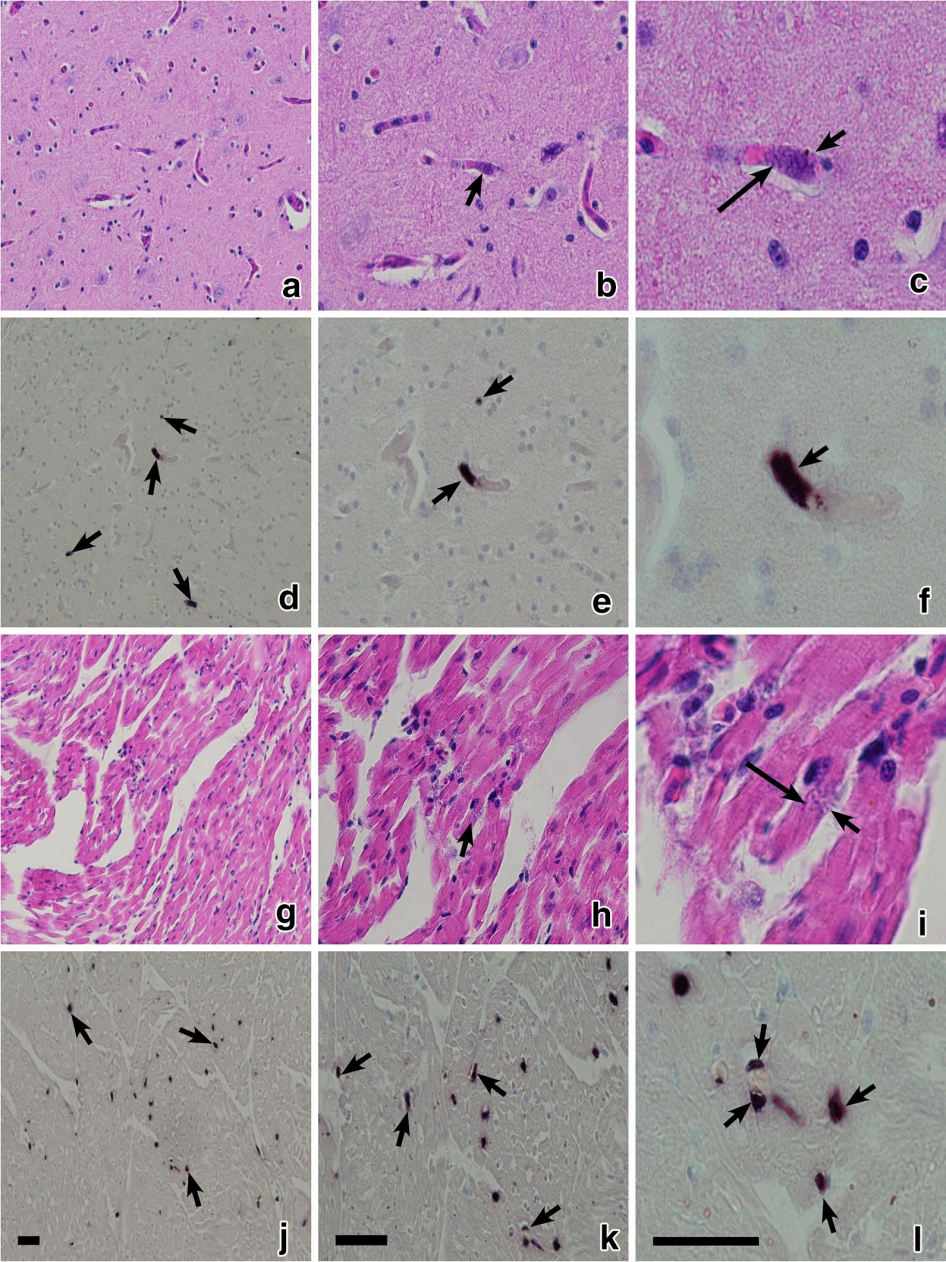
Phanerozoites of *Plasmodium (Giovannolaia) homocircumflexum* (cytochrome *b* lineage pCOLL4) in histological sections of the same organs stained with haematoxylin-eosin (**a**–**c**, **g**–**i**) and processed with chromogenic in situ hybridization (**d**–**f**, **j**–**l**): brain (**a**–**f**) and heart (**g**–**l**) of common crossbill *Loxia curvirostra.* Images of same preparations are given at low (×200; **a**, **d**, **g**, **j**), medium (×400; **b**, **e**, **h**, **k**) and high (×1000; **c**, **f**, **i**, **l**) magnifications. *Short arrows*: phanerozoites; *long arrows*: merozoites; *Scale bars* 25 μm

**Fig. 5:**
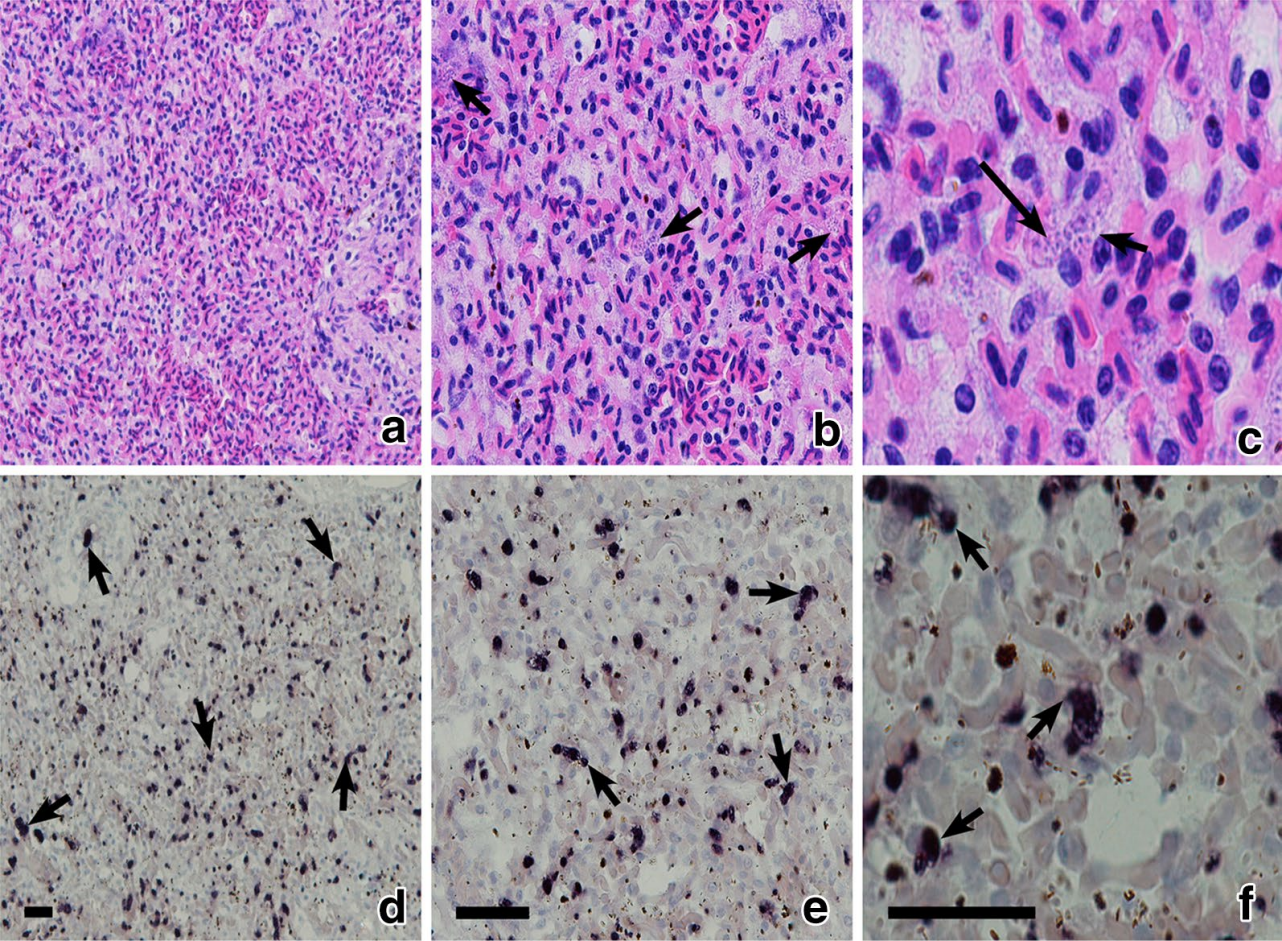
Phanerozoites of *Plasmodium (Giovannolaia) homocircumflexum* (cytochrome b lineage pCOLL4) in histological sections of same organs stained with hematoxylin-eosin (**a**–**c**) and processed with chromogenic in situ hybridization (**d**–**f**): lung (**a**–**f**) of common crossbill *Loxia curvirostra.* Images of same preparations are given at low (×200; **a**, **d**), medium (×400; **b**, **e**) and high (×1000; **c**, **f**) magnifications. *Short arrows*: phanerozoites; *long arrows*: merozoites; *Scale bars* 25 μm

Chromogenic ISH confirmed presence of phanerozoites in all examined preparations of the exposed birds. Positive ISH signals were readily visible in preparations of brain (Fig. 4d–f), heart (Fig. 4j–l), lungs (Fig. 5d–f), liver, spleen, kidney, and breast muscle of all exposed birds. They looked like black spots and could be easily counted (Fig. 4j–l) providing opportunity to estimate and compare intensity of phanerozoite infestation in different organs, which is difficult to do using microscopic examination of histological preparations stained with H&E (Figs. 4, 5). The negative controls used for the ISH assay did not give any positive signals.

At low (×200) magnification (Figs. 4a, d, g, j and 5a, d) it was nearly impossible to see phanerozoites in histological preparations stained with H&E, but they were readily visible in preparations treated for ISH (compare Figs. 4a, g and 5a with Figs. 4d, j and 5d, respectively). At medium magnification (×400) both histological (Figs. 4b, h and 5b) and ISH-treated preparations (Figs. 4e, k and 5e) allowed detection of phanerozoites, but it was difficult to determine their morphological features. At high magnification (×1000), both histological preparations (Figs. 4c, i and 5c) and those treated for ISH (Figs. 4f, l and 5f) allowed detection of phanerozoites, but structure of the parasites (shape of developing merozoites, morphology of the parasite envelope) cannot be recognized after ISH treatment (compare Fig. 4c with f). Chromogenic ISH markedly speeds up the search for phanerozoites, but morphological characters of the parasites cannot be determined using this technique.

## Discussion

The key results of this study are that: (1) *Plasmodium homocircumflexum* (pCOLL4) caused lethal malaria in three species of experimental passerine birds; (2) mortality occurs suddenly during decreased or even chronic stage of parasitaemia 4–6 weeks after exposure, most likely due to damage caused by phanerozoites; and (3) dynamics of parasitaemia were different, but patterns of phanerozoites development and tissue pathology were similar in all exposed birds. It is also important to note and worth discussion that ISH is an effective method in determining tissue stages of avian malaria parasites, but traditional histology remains essential for determining the structure of exoerythrocytic meronts.

*Plasmodium homocircumflexum* has been reported in several species of birds. The lineage pCOLL4 of this parasite along with the synonymous lineages pU12 (GenBank nr. DQ241519) and BOBO20085 (GenBank nr. KC867664) were detected in the red-backed shrike, domestic canary, collared flycatcher *Ficedula albicollis,* red-rumped warbling finch *Poospiza lateralis,* lark-like bushrunner *Coryphistera alaudina,* curve-billed reedhaunter *Limnornis curvirostris,* chopi blackbird *Gnorimopsar chopi,* chalk-browed mockingbird *Mimus saturninus,* diademed tanager *Stephanophorus diadematus,* bobolinks *Dolichonyx oryzivorus,* Eurasian siskin, common crossbill and common starling [14, 25–27, this study]. This parasite is virulent due to development of high parasitaemia in some hosts, but particularly because of its ability to produce numerous phanerozoites in many organs. This malarial infection kills domestic canaries [14] along with individuals belonging to three species of wild birds (this study), and it might be able to parasitize other bird species and be virulent in many of them, as is the case with the generalist lineages pSGS1 and pGRW4 of *Plasmodium relictum* and the lineage pGRW2 of *Plasmodium ashfordi* [18, 28–30]. Additional studies are needed for better understanding of virulence of this parasite in different bird species.

Transmission of *P. homocircumflexum* (pCOLL4) has not been reported in Europe, where this infection has been observed only in adult far-distance migrants after their arrival from African wintering grounds [14, 26]. It seems probable that European birds get infected away from their breeding areas. Lack of susceptible mosquito vectors might be an obstacle for this parasite’s transmission because sporogony was abortive in two common European mosquito species, *Culex pipiens* (forms *pipiens* and *molestus*) and *Aedes vexans* [14]. Vectors of *P. homocircumflexum* remain unknown. However, it is also difficult to rule out that transmission might occur at some sites in Europe, but infected birds are dying, as was the case during the experiments. Additional experimental studies combined with post-mortem examination of naturally infected dead birds collected in wildlife are needed for the better understanding of epidemiology of this malaria infection.

Patterns of development of *P. homocircumflexum* phanerozoites were similar in all exposed bird species (this study) and in domestic canaries [14], indicating similar life cycles and a probable high ability of this parasite to develop secondary exoerythrocytic meronts in many species of avian hosts. Blockage of circulation in brain capillaries (Fig. 2a, c) seems to be particularly dangerous because it can lead to ischaemic brain changes, which might cause cerebral paralysis symptoms, and can explain the observed sudden death of birds [4, 7]. Because mortality of all exposed birds was observed between 30 and 38 dpe, this is the probable period when phanerozoites could develop to an extent at which they start blocking the circulation in the brain. Additional experiments are needed for better understanding of this issue. Phanerozoites causing brain pathology due to blockage of circulation in the capillaries have been described in several avian malaria parasites: *Plasmodium gallinaceum, Plasmodium cathemerium, Plasmodium durae, Plasmodium lophurae, Plasmodium matutinum, Plasmodium octamerium*, and some other species [4].

Inflammatory changes in the brain cannot be expected in avian malaria, and this is supported by many reports [3, 4, 14]. Capillary blockage is the major pathological feature in most forms of avian malaria. In the present cases (Fig. 2 and Fig. 3), circulation disturbances can explain death of the birds (due to ischaemia of the brain), pericardial effusions (due to congestion of epicardial blood vessels) and in part also hepatomegaly and splenomegaly. In the latter two organs, infiltrations of inflammatory cells are an additional feature. Marked enlargements of the spleen and liver, as well as cardiomegaly with dark pericardial effusion were recorded at post-mortem examination in all birds and likely are important pathologies during this infection.

Brain lesions leading to an ischaemic brain changes are among the most severe conditions caused by the human malaria parasite *Plasmodium falciparum*, but the underlying functional pathology in humans and birds is different [4, 7, 31, 32]. Both in avian and human malaria, cerebral pathology occurs due to blockage of circulation in brain capillaries. However, birds are dying because of development of large phanerozoites, which follow the shape of brain capillaries eventually leading to the interruption of blood circulation (Fig. 2a, c). During *P. falciparum* malaria, severe pathology is caused by adherence of infected erythrocytes to the endothelial cells of microvascular blood vessels in the brain, leading to blockage of the circulation and resulting in ischaemic brain changes.

Mortality of birds due to *Plasmodium* infections have been reported both due to high parasitaemia and damage caused by phanerozoites [33, 34]. It is generally assumed that the decrease of parasitaemia after the acute stage of infection and the resulting chronic light parasitaemia indicate improved health in malaria infected birds [1, 35, 36]. This study shows that this is not always true because birds can die due to pathology caused by phanerozoites at chronic stage of infection when parasitaemia decreases (Fig. 1). The role of exoerythrocytic meronts in the pathology of birds is most likely underestimated during avian malaria because mortality might occur rapidly during light parasitaemia, and it is difficult to detect sick birds in wildlife. Recent studies using ISH method and DNA sequence data indicate that widespread *Plasmodium* parasite lineages are responsible for mortality in common European birds [9]; however, rates of mortality caused by malaria infections remain unknown in wildlife populations.

Better understanding of pathology caused by phanerozoites and other exoerythrocytic meronts of *Plasmodium* spp. is crucial for estimating of the true impact of these parasites on wild bird populations.

The number of “positive spots” after ISH treating is very high in some organs (Fig. 5d–f). That is in accordance with previous reports using this technique [8, 9] and certainly indicates parasites based on comparison with the negative controls, which were used both previously [8, 9] and in the present study. The application of a sensitive ISH assay for detection of exoerythrocytic meronts of avian malaria parasites and related haemosporidians markedly simplifies detection of affected organs, but it is not suitable for determining the structure of reported meronts. Histological methods using traditional staining provide additional data about morphology of tissue stages, which is important information for better understanding of the biology of malaria parasites. For example, two types of phanerozoites develop in *Plasmodium pinotti*: only roundish merozoites (micromerozoites) develop in the majority of phanerozoites, but phanerozoites containing elongate merozoites (macromerozoites) were also described [3, 37]. The role of these two types of merozoites remain unclear in the life cycle of *P. pinotti* and other avian malaria parasites, but this finding likely is important epidemiologically and might be related to peculiarities of persistence of malarial infections in avian hosts [4]. Ideally, both the traditional histology and ISH should be used in parallel in exoerythrocytic merogony research of haemosporidian parasites.

## Conclusion

*Plasmodium homocircumflexum* (pCOLL4) caused lethal malaria in at least four species of experimentally infected passerine birds due to marked damage of organs by phanerozoites. It is likely that this infection is markedly virulent in non-adapted wild birds and it worth more attention in bird conservation projects. The patterns of parasitaemia development were different, but patterns of phanerozoites development were similar in all tested exposed birds. Mainly, sudden mortality occurred during decreased or even light chronic parasitaemia stages in all exposed birds, indicating that the chronic parasitaemia is not necessarily an indication of improved health during avian malaria. In other words, solely testing of blood samples is an insufficient method to understand avian malaria virulence, which might be underestimated in many *Plasmodium* spp. due to lack of information about their exoerythrocytic development. During investigation of exoerythrocytic merogony of haemosporidians, application of an ISH method for detection of tissue meronts in bird organs, and then processing the ISH positive organs using traditional histological methods is recommended. Application of both these tools in parallel would speed up the search for tissue meronts and also provide information about morphological characters of the parasites and their host cells.
